# The role of perfusion and diffusion MRI in the management of patients affected by probable iNPH: A cohort-prospective preliminary study

**DOI:** 10.1186/2045-8118-12-S1-O23

**Published:** 2015-09-18

**Authors:** Francesco Tuniz, Maria Caterina Vescovi, Maria Cristina DeColle, Daniele Bagatto, Marta Maieron, Miran Skrap

**Affiliations:** 1 Azienda Ospedaliero Universitaria SM Misericordia, Italy

## Introduction

Two invasive tests are mainly used to select patients affected by NPH for surgery: measuring resistance to CSF outflow and Tap Test. Cerebral blood flow (CBF) have been demonstrated to be reduced in iNPH patients, mainly in basal ganglia (BG) and periventricular white matter (PVWM) regions. Perfusion MRI might be of value as a diagnostic and predictive tool. The rule of diffusion MRI in determining brain damage in PVWM and BG areas have been investigated. The aim of this study is to identify relationship between cerebral perfusion and microstructural damage of brain tissue measured by perfusion and diffusion MRI in PVWM and BG areas before and after tap-test and after sugery in patients who underwent VP shunt.

## Methods

23 patients were included in this study. MRI related rCBF and apparent diffusion coefficient (ADC) were calculated in all the cases. Regions of interest were located in PVWM and BG areas. Each patient underwent lumbar infusion test and tap test. After these tests were performed, patients have been clinically evaluated and another MRI was performed with the same protocol. Patients have been then divided into two groups: the first cohort that improved after Tap test, and the second one that did not. Only the first group underwent surgery and were clinically and radiological assessed again a month after VP shunt implantation. A descriptive statistical study was performed using non parametrical tests.

## Results

All the 13 patients surgically treated presented a clinical improvement after surgery; an significative increase in rCBF in both periventricular and basal ganglia regions after tap-test and surgery; a decrease in ADC values in periventricular region and an increase in ADC values in basal ganglia regions. The 10 negative patients shown a reduction in rCBF in both PVWM and BG after tap-test; a decrease in ADC values in PVWM and an increasing in ADC values in BG region.

## Conclusions

Since trend of rCBF acquired by perfusion MRI agreed with invasive tests results it could be considered a predictive and effective method in the management of patients with probable iNPH. Authors hypothesized decrease in ADC value in PVWM region as a reduction of transependimal edema; and increase in ADC value in basal ganglia area as a reduction in chronic cytotoxic edema due to chronic blood flow impairment.
